# Impact of Genetic Variation in Adrenergic Receptors on β-Blocker Effectiveness and Safety in Cardiovascular Disease Management: A Systematic Review

**DOI:** 10.3390/ph18040493

**Published:** 2025-03-28

**Authors:** Houwaida Abbes, Paula Soria-Chacartegui, Asma Omezzine, Francisco Abad-Santos, Pablo Zubiaur

**Affiliations:** 1 Clinical Pharmacology Department, Hospital Universitario de La Princesa, Instituto de Investigación Sanitaria La Princesa (IP), Universidad Autónoma de Madrid (UAM), 28049 Madrid, Spain; 2 Biochemistry Department, LR12SP11, Sahloul University Hospital, Sousse 4054, Tunisia; 3 Faculty of Pharmacy of Monastir, University of Monastir, Monastir 5000, Tunisia; 4 Centro de Investigación Biomédica en Red de Enfermedades Hepáticas y Digestivas (CIBERehd), Instituto de Salud Carlos III, 28029 Madrid, Spain

**Keywords:** β-blocker, adrenergic receptors, *ADRB1*, *ADRB2*, genetic variant, blockade

## Abstract

**Background/Objectives:** A systematic review was conducted to compile all the evidence on the impact of *ADRB1* and *ADRB2* genetic variants on the response to β-blockers, used for the management of cardiovascular diseases. **Methods:** After searching in PubMed, PharmGKB, and the Cochrane Central Register of Controlled Trials including terms related to these drugs, genes, and pathologies, 1182 articles were retrieved, 29 of which met the inclusion criteria. A β-adrenoreceptor (ADRB) blockade qualitative variable was inferred for all the associations between genetic variants and clinical phenotypes. **Results:** The relationship between *ADRB1* rs1801253 (G>C) C allele and higher receptor blockade showed a moderate overall level of evidence, reaching a high level on its relationship with higher reduction in the systolic (SBP) and diastolic blood pressure and heart rate (HR). The relationship between *ADRB1* rs1801252 (A>G) G allele and lower receptor blockade reached an overall high level of evidence, considering its impact on the reduction in the SBP, HR, left ventricular end-diastolic diameter, and incidence of major cardiovascular events. The relationship between *ADRB2* rs1042714 (G>C) C allele and lower receptor blockade reached a moderate overall level of evidence due to its impact on HR, pulmonary wedge pressure, and left ventricular ejection fraction response. The *ADRB2* rs1042713 (G>A) A allele was associated with higher receptor blockade and higher HR reduction with a low level of evidence. **Conclusions:** The genotyping of both *ADRB1* variants may be clinically useful; further investigation is required on the relevance of both *ADRB2* variants. Further research is warranted to determine the clinical usefulness of *ADRB* preemptive genotyping.

## 1. Introduction

Cardiovascular diseases (CVDs) are a primary cause of mortality and disability worldwide [1]. One of the most often prescribed classes of medications for the management of CVDs are β-adrenergic receptor antagonists (β-blockers) [2]. They show favorable effects on heart rate (HR) and blood pressure (BP) and are thereby indicated for the primary and secondary prevention of cardiovascular events in patients with ischemic heart disease, heart failure (HF), and hypertension [3], which reduces the risk for major cardiovascular events (MCEs) [4]. The response to β-blockers varies across individuals [5], which can be explained by the baseline demographic and clinical characteristics of patients, with an involvement of genetic factors. In fact, several works reporting associations between genetic variants and variability in β-blocker response have been reported to date, as described later in this manuscript. Genetic variations affecting β-blocker response could facilitate personalized treatment approaches, improving efficacy and reducing adverse reactions. The implementation of pharmacogenetic testing in clinical practice may help optimize drug selection and dosage, ultimately enhancing treatment success and patient outcomes.

The β1 and β2 adrenoceptors (ADRB1 and ADRB2, respectively), encoded by the *ADRB1* and *ADRB2* genes, are involved in the signaling processes of the sympathetic nervous system [6]. Catecholamines such as noradrenaline and adrenaline bind to and activate ADRB1, which results in increased HR and contractility [7]. Additionally, they induce the release of renin, which elevates BP [8]. While adrenaline activates ADRB2, causing the relaxation of the smooth muscles, which subsequently leads to an increase in metabolic processes, such as glycogenolysis, noradrenaline has minimal activity on ADRB2 [8,9]. β-blockers act as antagonists of ADRB1 and ADRB2 natural activator ligands, thereby causing a reduction in HR and BP [8,9]. β-blockers are classified into two groups: non-selective agents, which bind to β-1 and β-2 receptors, inducing antagonistic effects via both receptors, such as propranolol, while selective agents, such as atenolol or metoprolol, bind only to β-1 receptors, thereby conferring cardio-selectivity [8]. Some β-blockers, such as carvedilol, are additionally capable of blocking α-1 receptors [10], which differentiates them from selective β-blockers. This systematic review is focused on CVD treatment and, therefore, on β-receptor antagonism.

The Clinical Pharmacogenetics Implementation Consortium (CPIC) guideline examined the impact of genetic variations in the genes coding for ADRB1, ADRB2, for the cytochrome P450 2D6 (CYP2D6), the adrenoceptor α 2C (ADRA2C), and the G-protein kinase receptors 4 and 5 (GRK4 and GRK5) on the response to β-blockers [11]. Patients carrying the *ADRB1* rs1801252 (NG_012187.1: g.5231 A>G; NP_000675.1: p.Ser49Gly) A allele (Ser) or *ADRB1* rs1801253 (NG_012187.1: g.6251 G>C, NP_000675.1: p.Gly389Arg) C allele (Arg) are expected to exhibit a greater pharmacological response to β-blockers. The administration of low-dose β-blockers to patients with the C/C genotype (Arg/Arg) in *ADRB1* rs1801253 consistently resulted in a higher incidence of adverse drug reactions (ADRs) than in those patients with G/C (Gly/Arg) or G/G (Gly/Gly) genotypes; however, no genotype-specific differences in ADR incidence were observed at higher doses [11]. In contrast, the evidence on *ADRB2* variants is more limited, with fewer studies available in the literature compared to the *ADRB1* gene [11]. No clinical recommendations or prescribing information were provided in this guideline based on *ADRB1* or *ADRB2* genotypes. On the contrary, it includes metoprolol prescribing recommendations based on the *CYP2D6* genotype, the gene encoding for CYP2D6, an important metabolizing enzyme of several β-blockers [12].

CPIC and other scientific, academic, or medical organizations often examine the impact of genetic variation in relation to a single clinical event or clinical phenotype, such as exposure, safety, or efficacy, by systematically compiling all the available studies addressing such association. In the case of *ADRB* polymorphism, related to β-blocker pharmacodynamics, different clinical phenotypes can be associated with the receptor’s performance. Moreover, while the CPIC guideline provides valuable insights into genetic variation and β-blocker response, analyzing the degree of receptor blockade achieved with different genotypes could be a useful approach to enhance the understanding of these associations. It may help to clarify how the level of receptor activity influences treatment outcomes and could further inform about personalized β-blocker therapy. For instance, as observed with the *ADRB1* rs1801253 variant, associations between safety and efficacy tend to align, with higher efficacy often correlating with an increased risk of ADRs. The main ADRs associated with β-blocker treatment are bradycardia, hypotension, fatigue, and bronchospasm. It is hypothesized that this alignment occurs because the genetic variant causes an enhanced ADRB1 receptor blockade by β-blockers, which increases not only its efficacy but also the risk of ADRs. Although other systematic reviews on the relation between β-blockers response and genetic variation in ADRB1 and ADRB2 exist [9,13,14], to our knowledge, none of them integrated the individual associations into a single pharmacodynamic variable —the extent of receptor blockade.

The purpose of this systematic review was to compile and summarize all the available evidence on the impact of genetic variants in *ADRB1* and *ADRB2* on the efficacy and safety of β-blockers in CVD management. Furthermore, based on the associations between genetic variants and efficacy and safety signals, a variable denoted as ADRB blockade was inferred for a subsequent integrated analysis.

## 2. Materials and Methods

### 2.1. Search Strategy

A systematic review of the published literature by November 2024 was performed. Three electronic databases (i.e., PubMed, PharmGKB, and the Cochrane Central Register Of Controlled Trials (central)) were searched. Different combinations of Medical Subject Heading (MeSHs) terms were used, including: “*ADRB1*”, “*ADRB2*”, “beta adrenergic receptor gene”, “SNV”, “polymorphism”, “haplotype”, “beta blocker”, “beta blockade”, “cardiovascular disease”, “hypertension”, and “heart failure”, all of them combined using Boolean operators and adapted to each database. This systematic review was carried out in accordance with the “Preferred Reporting Items for Systematic Reviews and Meta-Analyses” (PRISMA) guidelines [15].

### 2.2. Study Selection and Inclusion

The inclusion criteria were as follows: articles written in English, performed on patients receiving β-blockers for the treatment of CVDs, and describing associations between *ADRB* genetic variants and pharmacotherapy outcomes. Reviews, in vitro studies, and studies conducted in languages other than English were excluded. Afterwards, an abstract and/or full-text check was performed if necessary to ensure compliance with inclusion criteria. Only studies for which the full text was available were included in this systematic review. When numerical data were not explicitly provided, studies were considered if they reported the direction of the association and statistical significance. An initial revision was performed to determine whether the obtained articles (n = 1182) met the inclusion criteria.

### 2.3. Level of Evidence

An “ADRB blockade” qualitative variable was inferred for all the associations between genetic variants and clinical phenotypes based on efficacy and safety parameters. *ADRB* variants related to higher efficacy (e.g., sharper BP decreases or lower dose requirements) or to a higher incidence of ADRs associated with the β-blocker mechanism of action (e.g., dizziness or hypotension) were classified as “higher ADRB blockade”. On the contrary, variants related to lower efficacy or to adverse events (AEs) involving the lack of effect of the drug (e.g., hypertension or tachycardia) were classified as “lower ADRB blockade”. A level of evidence was subsequently assigned to each variant for its overall relationship with ADRB blockade: the number of articles favoring higher and lower ADRB receptor blockade in the presence of each allele was counted. A ratio equal or superior to 3:1 of articles favoring higher (or lower) blockade was classified as high-level evidence; a ratio between 2:1 and 3:1, as moderate evidence; and a ratio below 2:1, as low evidence.

## 3. Results

**Figure 1:**
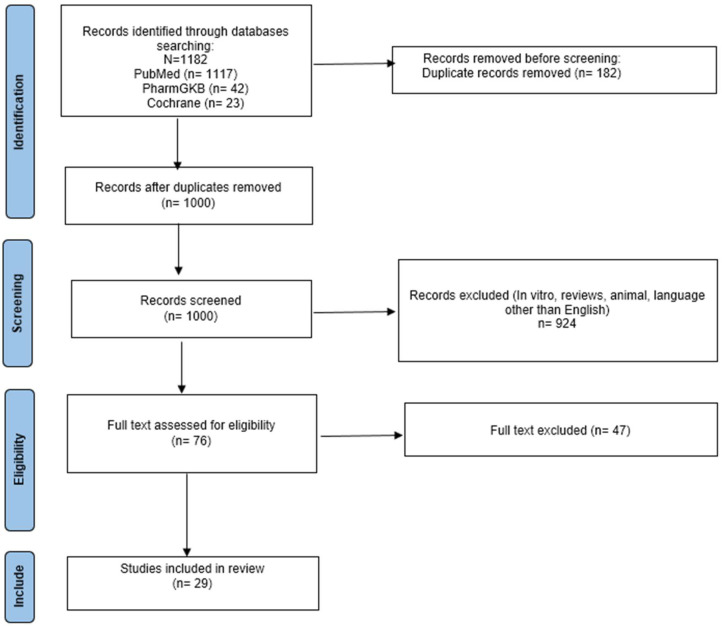
PRISMA flow chart of this systematic review.

The systematic search yielded a total of 1182 articles, 1117 from PubMed, 42 from PharmGKB, and 23 from Cochrane Central. Deduplication was manually performed in Microsoft Excel and it led to the removal of 182 duplicated articles. After title and abstract screening, 924 articles were removed, and the remaining 76 articles were fully examined. Finally, 29 articles were included in this systematic review (Figure 1); the remaining 47 were excluded because of the lack of a direct relationship between genotype and cardiovascular outcomes with the medication, or because the study did not address outcomes related to cardiovascular diseases, or did not perform comparisons between genotypes. In the CPIC guideline [11], 24 of these 29 articles were cited [16,17,18,19,20,21,22,23,24,25,26,27,28,29,30,31,32,33,34,35,36,37,38,39].

In the 29 included articles, four genetic variants located in *ADRB1* and *ADRB2* were identified. Two single nucleotide variants (SNVs) in the *ADRB1* gene were examined. The first variant was *ADRB1* rs1801253 (NG_012187.1: g.6251 G>C, NP_000675.1: p.Gly389Arg), included in 22 studies [16,17,18,19,20,21,22,23,24,25,26,27,28,29,30,31,32,33,34,40,41,42]. The second *ADRB1* variant was rs1801252 (NG_012187.1: g.5231 A>G; NP_000675.1: p.Ser49Gly), included in five studies [19,22,24,35,36]. Similarly, two SNVs in *ADRB2* were reported: rs1042714 (NG_016421.2: g.5318 G>C; NP_000015.2: p.Glu27Gln) in eight studies [7,34,37,38,39,43,44,45], and rs1042713 (NG_016421.2: g.5285 G>A; NP_000015.2: p.Gly16Arg) in two studies [44,45].

### 3.1. ADRB1 rs1801253 (NG_012187.1: g.6251 G>C, NP_000675.1: p. Gly389Arg)

A total of 19 studies [16,17,18,19,20,21,22,23,24,25,26,27,28,29,30,32,33,40,41] reported statistically significant associations between this variant and the efficacy of β-blockers, and four [31,32,34,42] with safety signals (Table 1).

Table 1 and Table 2 summarize all the studied clinical phenotypes along with the consolidated ADRB blockade information. The level of evidence for each individual clinical phenotype was calculated, as well as the overall level of evidence. The outcomes evaluating survival and all-cause mortality (ACM) were excluded from the analysis of the receptor blockade (Table 2), as these clinical phenotypes were considered too broad to analyze its relationship with receptor blockade. With a high level of evidence, higher SBP, DBP, and HR reduction were related to higher ADRB blockade, linked to the *ADRB1* rs1801253 C allele (Arg). The level of evidence between the remaining clinical phenotypes and ADRB1 blockade was low to moderate, with a moderate overall level of evidence (20:7 ratio).

### 3.2. ADRB1 rs1801252 (NG_012187.1: g.5231 A>G; NP_000675.1: p.Ser49Gly)

The search for this SNV encountered five articles describing efficacy [19,22,24,35,36] and none describing safety (Table 3).

Table 3 and Table 4 summarize all the studied clinical phenotypes along with the consolidated ADRB blockade information. The level of evidence for each individual clinical phenotype was calculated, as well as the overall level of evidence. With a moderate level of evidence, higher SBP reduction was related to higher ADRB blockade, linked to *ADRB1* rs1801252 A allele (Ser). The level of evidence between the remaining clinical phenotypes and ADRB1 blockade was low. However, overall, a high level of evidence in favor of *ADRB1* rs1801252 A allele (Ser) allele causing a higher ADRB1 blockade was obtained (4:1 ratio).

### 3.3. ADRB2 rs1042714 (NG_016421.2: g.5318 G>C; NP_000015.2: p.Glu27Gln)

The search for this SNV encountered eight results, with six describing efficacy [27,37,38,39,43,44] and two describing safety [34,45] (Table 5).

Table 5 and Table 6 summarize all the studied clinical phenotypes along with the consolidated ADRB blockade information. The level of evidence for each individual clinical phenotype was calculated, as well as the overall level of evidence. The outcomes evaluating the lipid parameters and survival were excluded from the analysis of the receptor blockade. The reason for this is that lipid metabolic alterations are not expected to occur through ADRB blockade, but through an alternative undesired pharmacodynamic effect; and because survival was considered a clinical endpoint too broad to deduct its relationship with ADRB blockade. With a moderate level of evidence, the increased left ventricular ejection fraction (LVEF), which is related to higher ADRB2 blockade, was linked to *ADRB2* rs1042714 G allele (Glu). The level of evidence between the remaining clinical phenotypes and ADRB2 blockade was low, with a moderate overall level of evidence.

### 3.4. ADRB2 rs1042713 (NG_016421.2: g.5285 G>A; NP_000015.2: p.Gly16Arg)

This SNV has been the subject of limited investigation. The systematic search encountered only two results, one evaluating efficacy [44] and another describing safety [45] (Table 7).

Table 7 and Table 8 summarize all the studied clinical phenotypes along with the consolidated ADRB blockade information. The level of evidence for each individual clinical phenotype was calculated, as well as the overall level of evidence. The level of evidence for the association between *ADRB2* rs1042713 and HR variations was assigned a moderate level of evidence, with carriers of the A allele (Arg) presenting higher receptor blockade and therefore higher reduction in this parameter compared to carriers of the G allele (Gly). The outcome evaluating ACM was excluded from the analysis of the receptor blockade, as this clinical phenotype was considered too broad to analyze its relationship with receptor blockade. Due to the limited literature available for this variant, the overall level of evidence assigned was low.

## 4. Discussion

According to the World Health Organization, CVD is the leading cause of mortality worldwide, and β-blockers remain the first-line medication for its management [46,47]. In cardiovascular diseases such as heart failure, β-adrenergic receptor downregulation and desensitization may attenuate the expected effects of *ADRB1* and *ADRB2* genetic variation on β-blocker response. However, to the best of our knowledge, no systematic review or guideline has attempted to synthesize all the evidence on how genetic variation on *ADRB1* and *ADRB2* impacts the efficacy and toxicity of β-blockers, particularly in relation to the degree of the receptor blockade. Furthermore, no review has sought to compile all the individual associations into a single pharmacodynamic variable, which is the enhanced or impaired receptor blockade. Consequently, this research is the first to address such a relation. After a comprehensive analysis of 29 articles, four genetic variants located in *ADRB1* and *ADRB2* were significantly associated with differences in β-blocker response.

### 4.1. ADRB1 Gene

The gene encoding for ADRB1 is located in the forward strand of the long arm of chromosome 10 (10q25.3) (GenBank: ID:153) [48]. *ADRB1* is predominately expressed in cardiac tissue, and in renal and adipose tissue [6].

#### 4.1.1. ADRB1 rs1801253 (NG_012187.1: g.6251 G>C, NP_000675.1: p.Gly389Arg)

The missense variant *ADRB1* p.Gly389Arg (g.6251 G>C, rs1801253) [49] is located in the cytoplasmic tail of the receptor, which is critical for its interaction with G-proteins and the subsequent signaling pathways [50]. The frequency of this genetic variant varies among populations [49]. Globally, the frequency of the G allele (Gly) and the C allele (Arg) are 29.07% and 70.93%, respectively [51]. In the European population, the C allele (Arg) shows a frequency of 68.50%, reaching a frequency of 80.5% in the American population and of 57% in the African population [51].

β-blockers exert their therapeutic effect by reducing BP; thus, a higher antihypertensive effect (i.e., higher BP reduction) is expected to be caused by a higher receptor blockade. The antihypertensive effect was measured by three different metrics, which were changes in SBP, in DBP, and in mean arterial pressure. Changes in SBP in response to selective β-blockers treatment were analyzed in six articles [17,19,21,22,23,40], and in five of them [17,19,21,23,40], the C allele (Arg) was associated with a greater reduction in this parameter compared to the G allele (Gly) after selective β-blocker treatment. Therefore, it can be proposed that this allele is responsible for a higher receptor blockade. Similarly, variations in DBP were measured in five articles [18,19,20,22,40], and in four of them [18,19,20,40], the C allele (Arg) was linked to a greater DBP decrease compared to the G allele (Gly) in response to selective [19,20,40] and no-selective [18] β-blocker treatment. In fact, Si D et al. [18] observed a 4-fold reduction with the non-selective β-blocker carvedilol, while Johnson JA et al. [20] reported a 2-fold reduction with the selective β-blocker metoprolol. A higher reduction in SBP and in DBP in the presence of the G allele (Gly) after the treatment with the selective β-blocker bisoprolol compared to the C allele (Arg) was only reported in the work of Suonsyrjä et al. [22]. Lastly, three studies examined the antihypertensive effect in terms of arterial pressure outcome [16,19,21]. In two of them [19,21], a higher reduction in mean arterial pressure was described in individuals carrying the C allele (Arg) compared to those carrying the G allele (Gly) after selective β-blocker (metoprolol or atenolol, respectively) treatment. However, Chen et al. [16] observed a lower arterial pressure decrease in carriers of the C allele (Arg) compared to those with the G allele (Gly) after the intake of the selective β-blocker metoprolol. Thus, the evidence supporting the association between the presence of the C allele (Arg) in *ADRB1* rs1801253 (g.6251 G>C; p.Gly389Arg), greater receptor blockade, and therefore, greater antihypertensive effect, is high (ratio 11:3). Consequently, it is proposed that patients heterozygous and/or homozygous for *ADRB1* rs1801253 G>C C allele (Arg) may require dose reductions compared to those with the G/G genotype. The CPIC guideline for β-blockers [11] reached a similar conclusion but did not recommend specific dose adjustments.

The second outcome assessed in this systematic review related to this genetic variant was the HR, reported in four articles [23,24,25,26]. β-blockers exert their therapeutic effect by reducing HR; thus, a higher HR reduction is expected to be related to higher receptor blockade. In three of these works [23,24,26], the presence of the C allele (Arg) was related to a greater reduction in HR compared to carriers of the G allele (Gly) during selective β-blocker treatment. In fact, Liu J et al. [23] demonstrated that this effect persisted both in exercise and resting conditions at the three dosage levels of metoprolol (75, 150, and 225 mg). However, the research by Rau et al. [25] reported the inverse effect; a lower HR was observed in carriers of the G allele (Gly) compared to carriers of the C allele (Arg) after treatment with the non-selective β-blocker carvedilol. Consequently, these results suggest that the presence of the C allele (Arg) is related to greater receptor blockade and higher HR reduction compared to the G allele (Gly) with a high level of evidence. This aligns with the potential relevance of *ADRB1* rs1801253 preemptive genotyping.

The therapeutic effect of β-blockers is also based on causing an increase in LVEF, which was the clinical outcome examined in two articles [27,28]. In the works of Metra et al. [27] and Luo et al. [28], a higher rise in LVEF was observed in the presence of the C allele (Arg) compared to the presence of the G allele (Gly) after treatment with the non-selective β-blocker carvedilol or with the selective β-blocker metoprolol, respectively. As a result, the association between the C allele (Arg) in *ADRB1* rs1801253, higher LVEF, and higher receptor blockade is supported by a moderate level of evidence, which is also supportive of a genotype-informed prescribing strategy based on this variant.

The fourth clinical outcome studied was the β-blocker dose requirements. This outcome was explored in two articles, and contradictory results were observed [29,30]. However, this is likely explained by the very limited research conducted to date addressing this outcome.

Efficacy was also analyzed in terms of cardiovascular mortality (CVM) and HF hospitalization in one article [32], which reported a lower incidence of both outcomes in carriers of the C allele (Arg) compared to noncarriers, when treated with high β-blocker doses. This finding further justifies the genotype-informed prescription of β-blockers.

Lastly, the relationship between the genetic variation in *ADRB1* and the incidence of MCE was analyzed in two investigations, and the results observed were contradictory [33,41], again probably due to the limited number of works addressing this clinical endpoint.

The relationship between this variant and ADR incidence was only reported in the work of Zaugg et al. [42], and no replication was, therefore, observed, for which this individual association showed a low level of evidence. Furthermore, the safety outcomes of ACM and survival were excluded from the analysis since they were considered too broad to deduct its association with receptor blockade [31,32,34].

In conclusion, the *ADRB1* rs1801253 C allele (Arg) is related to higher receptor blockade compared to the G allele (Gly), with an overall moderate level of evidence (ratio 20:7) and a high level of evidence for some of the studied clinical endpoints. Therefore, it is concluded that this variant may be clinically relevant. Further research is warranted to determine whether *ADRB1* rs1801253 preemptive genotyping is advisable to inform β-blocker prescription.

#### 4.1.2. ADRB1 rs1801252 (NG_012187.1: g.5231 A>G; NP_000675.1: p.Ser49Gly)

The missense variant *ADRB1* p.Ser49Gly (g.5231 A>G, rs1801252) [52] is located in the extracellular N-terminal domain, which is critical for its role in ligand binding and receptor stability [50]. The frequency of this genetic variant varies among populations [52]. Globally, the A allele shows a frequency of 87%, whereas the G allele shows a frequency of 13,5% [53]. The frequency of the G allele in the European population is 13%, reaching its highest frequency in the African population (25.8%) and its lowest in the South Asian population (10%) [53].

Both Liu et al. [19] and Suonsyrjä et al. [22] reported that carrying the A allele (Ser) was related to a greater SBP reduction compared to carrying the G allele (Gly) during selective β-blocker treatment (metoprolol and bisoprolol, respectively). As previously mentioned, a sharper decrease in BP is expected to be observed when there is a higher receptor blockade. Thus, the association between the A allele (Ser) in *ADRB1* rs1801252, a higher receptor blockade, and a greater SBP reduction is supported by a moderate level of evidence.

The therapeutic effect of β-blockers also relies on causing a reduction in HR, LVEDD, and MCE incidence. Each of these outcomes was analyzed in a single work [24,35,36]. The A allele (Ser) was associated with a better HR response and a lower MCE incidence compared to the G allele (Gly) after β-blocker treatment [24,36]. On the contrary, in the work of Terra et al. [35], an LVEDD reduction was observed in the presence of the G allele (Gly), whereas carrying the A allele (Ser) was related to an increase in LVEDD. However, since none of these outcomes were replicated, these individual associations showed a low level of evidence. To our knowledge, no associations between variations in safety and this SNV were reported in the literature.

In conclusion, the *ADRB1 rs1801252* A allele (Ser) was proposed to cause a higher receptor blockade and therefore higher efficacy than the G allele (Gly), with a high level of evidence (ratio 4:1), manifested as improved SBP and HR response and reduced MCE incidence. These results are consistent with those reported in the CPIC guideline, in which the A allele (Ser) was associated with a better response to β-blocker treatment [11]. This variant, along with *ADRB1* rs1801253, contributes to the pool of potential pharmacogenetic biomarkers that may be worth genotyping preemptively for β-blocker prescription.

### 4.2. β-2 Adrenergic Receptor (ADRB2)

The gene encoding for ADRB2 is located in the forward strand of the long arm of chromosome 5 (5q32) (GenBank: ID:154) [54]. *ADRB2* is primarily expressed in the pulmonary system, vascular smooth muscle, and central nervous system [55].

#### 4.2.1. ADRB2 rs1042714 (NG_016421.2: g.5318 G>C; NP_000015.2: p.Glu27Gln)

The missense variant ADRB2 p.Glu27Gln (g.5318 G>C, rs1042714) [56] is located in the extracellular domain of the receptor [57], and it is thought to modify receptor function and regulation. The frequency of this genetic variant varies among populations [56]. Globally, the frequency of the G allele and the C allele are 39.72% and 60.28%, respectively [58]. The frequency of the C allele in the European population is 59%, where it reaches its lower frequency [58]. The highest frequency of the C allele is observed in the East Asian population (92.7%) [58].

Shahin et al. [44] observed that patients carrying the G allele (Glu) showed a greater HR reduction after atenolol or metoprolol treatment compared to those carrying the C allele (Gln). Due to the lack of replication, this association was assigned a low level of evidence.

Furthermore, both Kaye et al. [39] and Metra et al. [27] reported a greater LVEF increase in carriers of the G allele (Glu) compared to carriers of the C allele (Gln) during carvedilol treatment and therefore, a higher receptor blockade in the presence of the G allele (Glu). Moreover, the decline in pulmonary wedge pressure, a therapeutic effect associated with β-blocker treatment, was investigated by Metra et al. [27]. It was reported that individuals carrying the G allele (Glu) presented a larger decline in pulmonary wedge pressure both at rest and at peak exercise compared to individuals with the C allele (Gln). Since two different evaluations of LVEF and pulmonary wedge pressure were available, the level of evidence supporting the association between the G allele (Glu) in *ADRB2* rs1042714, a higher receptor blockade, and a greater improvement in pulmonary wedge pressure and in LVEF, was considered moderate.

The evaluation of lipid parameters was another outcome related to this SNV, which was analyzed in three articles [37,38,43]. However, it was excluded from the analysis since these metabolic effects are not mediated by the blockade of ADRB. Moreover, the only safety-related outcome identified in relation to this SNV was survival, evaluated in two studies [34,45]. Similarly, it was excluded from the analysis of the ADRB blockade since it was considered an outcome too broad to deduct its association with receptor blockade.

Due to the limited literature available, the *ADRB2* rs1042714 G allele (Glu) was associated with higher receptor blockade with a moderate level of evidence, considering its effect on HR and pulmonary wedge pressure reduction and LVEF increase, compared to the C allele (Gln). Further research is required to analyze the relevance of this variant as a preemptive biomarker in β-blocker therapy. Moreover, its impact on the response to β-2 agonists, crucial in asthma management, should also be addressed.

#### 4.2.2. ADRB2 rs1042713 (NG_016421.2: g.5285 G>A; NP_000015.2: p.Gly16Arg)

The missense variant ADRB2 p.Gly16Arg (g.5285 G>A, rs1042713) [59] is located in the extracellular domain of the receptor [57], and it affects how the receptor responds to agonists and antagonists. The frequency of this genetic variant varies among populations [59]. The G allele shows a global frequency of 61.20%, whereas the A allele shows a global frequency of 38.80% [60]. The frequency of the A allele reaches in the European Population its lowest frequency (37.43%) and its highest frequency is reached in the Asian population (58.32%) [60].

The studies regarding this genetic variant were limited. Shahin MH et al. work [44] described the association between the presence of the A allele (Arg) and a better improvement in HR compared to the G allele (Gly) after atenolol treatment, which was observed in two different clinical trials. The search for safety also yielded only one article in which an association between this genetic variant and ACM was described [45]. However, this association was excluded from the analysis due to the breadth of the clinical endpoint. Due to the limited literature available for this variant, the overall level of evidence of the association between the A allele (Arg) in *ADRB2* rs1042713 and higher receptor blockade compared to the G allele (Gly) was assigned a low level of evidence (ratio 2:0). Further research should be performed to analyze the clinical relevance of this variant and its utility as a pharmacogenetic biomarker in β-blocker treatment. Furthermore, its impact on the response to β-2 agonists, crucial in asthma management, should also be addressed.

Moreover, further investigation exploring other genetic variants located in these genes is required, as other unknown SNVs affecting β-blocker response that might be potential pharmacogenetic biomarkers may also exist.

This systematic review has several limitations, such as the inclusion of different kinds of studies (randomized controlled trial, retrospective, prospective, etc.) which may hamper results comparison. Furthermore, the presence of confounding factors, such as concomitant medications, comorbidities, and differences in treatment adherence, may also have influenced the observed associations in each article. Additionally, while our review focused on well-characterized genetic variants in *ADRB1* and *ADRB2*, we did not thoroughly investigate other potential contributions to β-blocker responsiveness, such as rare genetic variants, gene–gene interactions, epigenetic alterations, and environmental variables. Future research incorporating multi-omics approaches may provide a more comprehensive understanding of pharmacogenetic influences on β-blocker therapy. Another limitation of this study is that most of the papers examined did not provide *CYP2D6* genotyping data. Because CYP2D6 is an important enzyme in the metabolism of β-blockers, interindividual variability in CYP2D6 activity may contribute to drug response disparities. Future research should include *CYP2D6* genotyping to improve the accuracy of pharmacogenetic predictions and clinical application. Despite these limitations, this review provides significant insights into the pharmacogenetics of β-blockers and highlights key areas for future investigation. By advancing our understanding of these mechanisms, this approach could support future guidelines in refining pharmacogenetic-based recommendations for clinical practice.

## 5. Conclusions

This systematic review identified four SNVs in the *ADRB* genes related to variations in diverse efficacy and safety outcomes. The relationship between the A allele (Ser) in *ADRB1* rs1801252 and a higher receptor blockade reached a high level of evidence. The relationship between the C allele (Arg) in *ADRB1* rs1801253, the G allele (Glu) in *ADRB2* rs1042714, and a higher receptor blockade reached a moderate level of evidence. The relationship between the A allele (Arg) in *ADRB2* rs1042713 and a higher receptor blockade reached a low level of evidence. The genotyping of both *ADRB1* variants may be clinically useful, whereas further investigation is required on the relevance of *ADRB2* variants. Future research is crucial to determine the usefulness of *ADRB1* genotyping and genotype-informed β-blocker prescription in the clinical setting.

## Figures and Tables

**Table 1:** Association between *ADRB1* rs1801253 (g.6251 G>C; p.Gly389Arg) and β-blocker efficacy and safety.

Author (Year)	Study Type	Population	Drug Used (Monotherapy or Combination)	Outcome	Association	Genotyping of *CYP2D6*	Significance	ADRB Blockade
Efficacy								
Chen L et al. (2018) [16]	Retrospective study	261 hypertensive patients	Metoprolol (monotherapy)	Antihypertensive effect	Patients with G/G genotype (Gly/Gly) showed higher metoprolol antihypertensive effects compared to patients with G/C genotype (Gly/Arg) (numeric values not provided).	Yes	*p* = 0.027	G (Gly): higher C (Arg): lower
Wu D et al. (2015) [17]	Retrospective study	93 hypertensive patients	Metoprolol (monotherapy)	Antihypertensive effect	Patients with the C/C genotype (Arg/Arg) showed greater SBP reduction after treatment compared to those with the G/C genotype (Gly/Arg) (127 mmHg vs. 132 mmHg).	Yes	*p* < 0.05	G (Gly): lower C (Arg): higher
Si D et al. (2014) [18]	Prospective study	87 hypertensive patients	Carvedilol (monotherapy)	Antihypertensive effect	Patients with the C/C genotype (Arg/Arg) showed a four-fold greater reduction in DBP with carvedilol compared to those with the G/G genotype (Gly/Gly) (10.61 mmHg vs. 2.62 mmHg).	No	*p* = 0.013	G (Gly): lower C (Arg): higher
Liu J et al. (2006) [19]	Prospective clinical trial	223 hypertensive patients	Metoprolol (monotherapy)	Antihypertensive effect	Patients with G/G (Gly/Gly) or G/C (Gly/Arg) genotype showed a lower reduction in SBP after metoprolol treatment compared to those with C/C genotype (Arg/Arg) (G/G: 1.10% ± 1.50%, G/C: 2.80% ± 4.70%, C/C: 10.40% ± 4.00%).	No	*p* < 0.001 (G/G vs. C/C) *p* = 0.001 (G/C vs. C/C)	G (Gly): lower C (Arg): higher
					Patients with C/C (Arg/Arg) or G/C (Gly/Arg) genotype showed a greater reduction in DBP after metoprolol treatment compared to those with G/G genotype (Gly/Gly) (C/C: 6.10% ± 4.30%, G/C: 2.20% ± 4.20%, G/G: 0.90% ± 4.00%).		*p* < 0.001	G (Gly): lower C (Arg): higher
					Patients with G/G genotype (Gly/Gly) showed a lower reduction in mean arterial pressure after metoprolol treatment compared to those with G/C (Gly/Arg) or C/C (Arg/Arg) genotype (G/G: 1.00% ± 2.50%; G/C: 2.50% ± 3.00%, C/C: 8.10% ± 3.50%).		*p* < 0.001	G (Gly): lower C (Arg): higher
Johnson JA et al. (2003) [20]	Prospective clinical trial	40 hypertensive patients	Metoprolol (monotherapy)	Antihypertensive effect	Patients with C/C genotype (Arg/Arg) showed a two-fold greater reduction in 24 h DBP after metoprolol treatment compared to those with G/G (Gly/Gly) or G/C (Gly/Arg) genotype (−12.00% ± 8.60% vs. −5.10% 7.80%). *	No	*p* = 0.012	G (Gly): lower C (Arg): higher
					Patients with the C/C genotype (Arg/Arg) showed a 6.5 mmHg greater absolute reduction in 24 h DBP compared to those with G/G (Gly/Gly) or G/C (Gly/Arg) genotype (95% CI −1.70 to −11.30 mmHg). *		*p* = 0.009	G (Gly): lower C (Arg): higher
					Patients with C/C genotype (Arg/Arg) showed an 8.6 mmHg greater reduction in daytime DBP compared to those with G/G (Gly/Gly) or G/C (Gly/Arg) genotype (95% CI −3.50 to −13.60 mmHg). *		*p =* 0.0014	G (Gly): lower C (Arg): higher
Sofowora GG et al. (2003) [21]	Prospective study	400 healthy volunteers	Atenolol (monotherapy)	Antihypertensive effect	Patients with the G/G genotype (Gly/Gly) showed a lower reduction in resting SBP in response to atenolol compared to those with the C/C genotype (Arg/Arg) (G/G: 0.20 ± 1.70 mm Hg, C/C: 8.70 ± 1.30 mm Hg).	No	*p* = 0.001	G (Gly): lower C (Arg): higher
					Patients with the G/G (Gly/Gly) genotype showed a lower reduction in mean arterial pressure in response to atenolol compared to those with the C/C genotype (Arg/Arg) (G/G: 2.00 ± 1.70 mmHg, C/C: 7.20 ± 1.00 mmHg).		*p* = 0.009	G (Gly): lower C (Arg): higher
Suonsyrjä T et al. (2010) [22]	Randomized Controlled Trial	233 hypertensive patients	Bisoprolol (monotherapy)	Antihypertensive effect	Patients with the G/G genotype (Gly/Gly) showed a greater SBP reduction in response to bisoprolol compared to those with the C/C genotype (Arg/Arg) (−15.80 mmHg (−16.80, −9.10) vs. −10.30 mmHg (−14.30, −6.40)).	No	*p* = 0.003	G (Gly): higher C (Arg): lower
					Patients with the G/G genotype (Gly/Gly) showed a greater DBP reduction in response to bisoprolol compared to those with the C/C genotype (Arg/Arg) (−11.20 mmHg (−14.40, −9.00) vs. −7.90 mmHg (−10.60, −5.40)).		*p* = 0.003	G (Gly): higher C (Arg): lower
Fayed MS et al. (2023) [40]	Prospective study	77 new onset acute coronary syndrome patients	Bisoprolol (combination with ACEi, nitroglycerin, spironolactone, loop diuretics, clopidogrel, aspirin, and statins)	Antihypertensive effect	Patients with the C/C genotype (Arg/Arg) showed a greater DBP reduction compared to those with the G/G (Gly/Gly) or G/C (Gly/Arg) genotype (−9.5% ± 9.7% vs. −0.80% ± 11.5%). ^$^	No	*p* = 0.00015	G (Gly): lower C (Arg): higher
					Patients with the C/C genotype (Arg/Arg) showed an 8 mm Hg greater absolute reduction in 24 h DBP compared to those with the G/G (Gly/Gly) or G/C (Gly/Arg) genotype (95% CI: −8.8 to −7.3 mm Hg). ^$^		*p* = 0.00012	G (Gly): lower C (Arg): higher
					Patients with G/G (Gly/Gly) and G/C (Gly/Arg) showed a lower reduction in SBP compared to those with the C/C genotype (Arg/Arg) (−0.76% ± 8.7% vs. −8.5% ± 7.8%). ^@^		*p* = 0.000218	G (Gly): lower C (Arg): higher
					Patients with the G/G genotype (Gly/Gly) showed a −9.6 mmHg lower absolute reduction in 24 h SBP compared to those with the C/C (Arg/Arg) and G/C (Gly/Arg) genotypes (95% CI: −10.5 to −8.7 mmHg). ^@^		*p* = 0.00012	G(Gly): lower C (Arg): higher
Liu J et al. (2003) [23]	Prospective study	123 male volunteers	Metoprolol (monotherapy)	Antihypertensive effect	Volunteers with the G/G (Gly/Gly) or G/C (Gly/Arg) genotype showed a lower SBP reduction at the 3 dosage levels of metoprolol (75, 150, 225 mg) compared to those with the C/C genotype (Arg/Arg) (4.60% ± 0.50% vs. 5.90% ±0.70%, 6.00% ± 0.80% vs. 9.20% ±1.00%, 9.90%± 0.90% vs. 11.60% ± 1.20%, respectively)	No	*p* = 0.011	G (Gly): lower C (Arg): higher
				HR	Volunteers with the C/C genotype (Arg/Arg) showed a greater reduction in resting HR at the 3 dosage levels of metoprolol (75, 150, 225 mg) compared to those with the G/G (Gly/Gly) or G/C (Gly/Arg) genotype (6.30% ± 0.80% vs. 4.10% ± 0.70%, 10.10% ± 1.00% vs. 6.20% ± 1.10%, and 14.40% ±1.40% vs. 10.90% ±1.30%, respectively).		*p* = 0.008	G (Gly): lower C (Arg): higher
				HR	Volunteers with the C/C genotype (Arg/Arg) showed a greater reduction in exercise HR at the 3 dosage levels of metoprolol (75, 150, 225 mg) compared to those with the G/G (Gly/Gly) or G/C (Gly/Arg) genotype (8.90% 0.50% vs. 6.60% 0.70%, 14.00% 0.90% vs. 11.70% 1.00%, and 20.10% 1.50% vs. 16.40% 1.30%, respectively).		*p* = 0.017	G (Gly): lower C (Arg): higher
Cotarlan V et al. (2013) [24]	Prospective study	201 patients scheduled for non-invasive coronary computed tomographic angiography	Metoprolol (monotherapy)	HR	Patients with the G/G genotype (Gly/Gly) showed a greater percentage of non-response rate (HR > 60 beats/min) compared to those with the C/C (Arg/Arg) or G/C (Gly/Arg) genotype (45% vs. 17%).	No	*p =* 0.020	G (Gly): lower C (Arg): higher
Rau T et al. (2012) [25]	Prospective study	876 patients with HF	Bisoprolol and carvedilol (monotherapy)	HR	Patients with the C/C genotype (Arg/Arg) showed greater HR during titration in carvedilol treatment compared to those with the G/G (Gly/Gly) and G/C (Gly/Arg) genotypes (90 beats/min vs. 77 beats/min).	No	*p* < 0.0001	G (Gly): higher C (Arg): lower
Kurnik D et al. (2009) [26]	Prospective study	154 healthy volunteers	Atenolol (monotherapy)	HR	Volunteers with the C/C (Arg/Arg) or G/C (Arg/Gly) genotype showed greater HR reduction after atenolol treatment compared to those with the G/G (Gly/Gly) genotype (numeric values not provided).	No	95% CI:11.7 (3.80–19.5) *p* = 0.040	G (Gly): lower C (Arg): higher
Metra M et al. (2010) [27]	Prospective study	183 patients with HF	Carvedilol (combination with ACEi, diuretics, or aldosterone antagonists)	LVEF	Patients with the C/C (Arg/Arg) or G/C (Gly/Arg) genotype showed a higher rise in LVEF after treatment compared to those with the G/G genotype (Gly/Gly) (C/C: 7.80 ± 7.60%, G/C: 9.00 ± 11.40%, C/C: 4.10 ± 7.60% units).	No	*p* = 0.0847 (G/G vs. C/C) *p* = 0.1058 (G/C vs. C/C)	G (Gly): lower C (Arg): higher
Luo M et al. (2007) [28]	Prospective study	156 patients with HF	Metoprolol (combination with digitalis, ACEi, and diuretics)	LVEF	Patients with the C/C genotype (Arg/Arg) showed a higher difference in LVEF before and after metoprolol treatment compared to those with the G/C genotype (Gly/Arg) (4.60% ± 2.98% vs. 1.90% ± 2.04%).	No	*p* = 0.027	G (Gly): lower C (Arg): higher
Baudhuin LM et al. (2010) [29]	Retrospective study	93 patients with HF	Metoprolol and carvedilol (combination with ACEIs, ARBs, and diuretics)	Dose–response	Patients with the G/G genotype (Gly/Gly) needed an approximately 25 mg higher carvedilol daily dose compared to those with the G/C (Gly/Arg) genotype.	Yes	*p* = 0.020	G (Gly): lower C (Arg): higher
Lee HY et al. (2016) [30]	Prospective study	100 patients with HF	Bisoprolol (combination with ACEi, ARB, Spironolactone, loop diuretics, and digoxin aspirin)	Dose–response	Patients with the C/C (Arg/Arg) genotype required higher doses of bisoprolol compared to those with the G/G (Gly/Gly) or G/C (Gly/Arg) genotype (5.26 ± 2.62 mg vs. 3.96 ± 2.05 mg).	No	*p* = 0.022	G (Gly): higher C (Arg): lower
Parikh KS et al. (2018) [32]	Retrospective analysis of RCTs (BEST, HF-ACTION)	1040 patients under β-blocker treatment	Bucindolol (monotherapy)	CVM/HF hospitalization	Patients with the C/C genotype (Arg/Arg) showed a higher CVM/HF hospitalization reduction at higher β-blocker doses compared to those with the G/G (Gly/Gly) or G/C (Gly/Arg) genotype (C/C: 24%, G/G + G/C:32%). There were no significant differences for no/low-dose β-blockers (C/C: 35%, G/G + G/C:34%).	No	*p* = 0.026	G (Gly): lower C (Arg): higher
Biolo A et al. (2008) [33]	Prospective study	201 patients with HF	Metoprolol and carvedilol (combination with ACEi, diuretics, and spironolactone)	MCE	Patients with the C/C genotype (Arg/Arg) showed a higher prevalence of non-sustained ventricular tachycardia compared to those with the G/G genotype (Gly/Gly) (48% vs. 17%).	No	*p* = 0.015	G (Gly): higher C (Arg): lower
Aleong RG et al. (2013) [41]	Randomized controlled clinical trial	1040 patients with HF	Bucindilol (combination with digoxine)	MCE	Patients with the C/C genotype (Arg/Arg) showed a lower incidence of new-onset arterial fibrillation in bucindolol treatment compared to those with the G/G (Gly/Gly) or G/C (Gly/Arg) genotype.	No	Hazard Ratio = 0.26 [95% CI: 0.12–0.57], *p* = 0.0003	G (Gly): lower C (Arg): higher
Safety								
Fiuzat M et al. (2013) [31]	Randomized, multicenter trial	902 patients under β-blocker treatment	Carvedilol and metoprolol(combination with loop diuretics)	ACM	Patients with the C/C genotype (Arg/Arg) receiving low-dose β-blockers showed a two-fold higher risk of death compared to those receiving high doses. There were no significant differences in risk between patients receiving low vs. high dose β-blockers among patients with the G/G genotype (Gly/Gly).	No	Hazard Ratio = 2.09; *p* = 0.015 Hazard Ratio = 0.91; *p* = 0.73	NA
Parikh KS et al. (2018) [32]	Retrospective analysis of RCTs (BEST, HF-ACTION)	1040 patients under β-blocker treatment	Bucindolol (monotherapy)	ACM	Patients with the C/C genotype (Arg/Arg) showed a 46% ACM reduction at higher bucindolol doses compared to those with the G/G (Gly/Gly) or G/C (Gly/Arg) genotype (C/C: 10%, G/G + G/C: 19%). There were no significant differences for no/low-dose bucindolol (C/C:21%, G/G + G/C:20%).	No	Hazard Ratio= 0.54; *p* = 0.018	NA
		957 patients with HF	Various beta-blockers: carvedilol, metoprolol, bisoprolol, and atenolol (combination with ACEIs, ARBs, and Aldosterone Receptor)	ACM	Patients with the C/C genotype (Arg/Arg) showed a greater ACM at lower β-blocker doses compared to those with the G/G (Gly/Gly) or G/C (Gly/Arg) genotype (C/C:21%, G/G + G/C:14%). There were no significant differences for high-dose β-blockers (C/C:10%, G/G + G/C:13%).		Hazard Ratio = 0.83; *p* = 0.015	NA
Zaugg M et al. (2007) [42]	Double-blinded, placebo-controlled, multicenter trial	224 patients undergoing surgery with a spinal block	Bisoprolol (combination with Ca2 antagonists, diuretics, ACEi, ARBs, nitrates, and statins)	Adverse events	Patients with the G/G genotype (Gly/Gly) showed a higher number of adverse events compared to those with the C/C genotype (Arg/Arg) (24 of 74 [32.40%] vs. 21 of 112 [18.70%]).	No	Hazard Ratio = 1.87 [95% CI: 1.04–3.35] *p* = 0.040	G (Gly): higher C (Arg): lower
Guerra LA et al. (2022) [34]	Retrospective study	308 patients with HF	Metoprolol and carvedilol (combination with ACEIs, ARBs, and diuretics)	Survival	Patients with the C/C genotype (Arg/Arg) showed a higher survival rate at higher β-blocker doses compared to those with the G/G (Gly/Gly) genotype.	No	*p* = 0.023	NA

SBP: systolic blood pressure; DBP: diastolic blood pressure; HR: heart rate; HF: heart failure; LVEF: left ventricular ejection fraction; ACM: all-cause mortality; CVM: cardiovascular mortality; MCE: major cardiovascular events; RCT: randomized controlled trial; ACEI: angiotensin-converting enzyme inhibitor; ARBs: angiotensin receptor blockers; CI: confidence interval; receptor blockade: the degree of β-receptor blockade. *^, $, @^: these results were considered a unique evaluation; therefore, they were only counted once. All-cause mortality and survival were excluded from this analysis since they were considered to be clinical outcomes too broad to analyze their relationship with receptor blockade.

**Table 2:** Outcomes, degree of receptor blockade, and evidence levels for *ADRB1* rs1801253 (p.Gly389Arg; g.6251 G>C) variant.

Outcome	Total Number of Articles	Outcome Variation Associated with Higher Receptor Blockade	Higher Blockade with C Allele (Arg)	Higher Blockade with G Allele (Gly)	Level of Evidence
Arterial pressure	3	Arterial pressure reduction	[19,21]	[16]	Moderate (2:1)
SBP	6	SBP reduction	[17,19,21,23,40]	[22]	High (5:1)
DBP	5	DBP reduction	[18,19,20,40]	[22]	High (4:1)
HR	5 *	HR reduction	[23,24,26]	[25]	High (4:1)
LVEF	2	LVEF increase	[27,28]		Moderate (2:0)
Dose requirements	2	Dose reduction	[29]	[30]	Low (1:1)
CVM and heart failure hospitalization	1	Lower mortality and hospitalization	[32]		Low (1:0)
MCE	2	Lower incidence	[41]	[33]	Low (1:1)
Adverse events	1	Higher incidence		[42]	Low (0:1)
Overall	27 ^$^		20	7	Moderate (20:7)

SBP: systolic blood pressure; DBP: diastolic blood pressure; HR: heart rate; LVEF: left ventricular ejection fraction; CVM: cardiovascular mortality; MCE: major cardiovascular events. * two independent results are presented in the same article; thus, the corresponding article was counted twice (Liu J et al. (2003) [23]). All-cause mortality and survival were excluded from this analysis since they were considered to be clinical outcomes too broad to analyze their relationship with receptor blockade. ^$^: results related to different outcomes were presented in some articles, which is why the overall number of articles is 27 and not 22.

**Table 3:** Association between *ADRB1* rs1801252 (g.5231 A>G; p.Ser49Gly) and β-blocker efficacy.

Author (Year)	Study Type	Population	Drug Used (Monotherapy or Combination)	Outcome	Association	Genotyping of *CYP2D6*	Significance	Receptor Blockade
Liu J et al. (2006) [19]	Prospective clinical trial	223 hypertensive patients	Metoprolol (monotherapy)	Antihypertensive effect	Patients with the A/A genotype (Ser/Ser) showed a greater SBP reduction in response to metoprolol compared to those with the A/G genotype (Ser/Gly) (8.40% ± 3.20% vs. 5.30% ± 5.20%).	No	*p* = 0.047	A (Ser): higher G (Gly): lower
Suonsyrjä T et al. (2010) [22]	RCT	233 hypertensive men	Bisoprolol (monotherapy)	Antihypertensive effect	Patients with the A/A genotype (Ser/Ser) showed a greater SBP reduction in response to bisoprolol compared to those with the A/G genotype (Ser/Gly) (−11.50 (−15.50, −7.00) mmHg vs. −9.90 (−13.40, −6.20) mmHg).	No	*p* = 0.04, *p* = 0.02 (Mann–Whitney U test and multivariate analysis)	A (Ser): higher G (Gly): lower
Cotarlan V et al. (2013) [24]	Prospective study	201 patients scheduled for non-invasive coronary computed tomographic angiography	Metoprolol (monotherapy)	HR	Patients with the G/G (Gly/Gly) or G/A (Ser/Gly) genotype showed a higher percentage of non-response rate (HR > 60 beats/min) compared to those with the A/A genotype (Ser/Ser) (29% vs. 15%).	No	*p =* 0.037	A (Ser): higher G (Gly): lower
Terraa SG et al. (2005) [35]	Prospective study	61 β-blocker naïve patients with systolic HF	Metoprolol (combination with ACEi, ARBs, furosemide, digoxin, spironolactone, or antiplatelet therapy)	LVEDD	Patients with G/G (Gly/Gly) and G/A (Ser/Gly) showed an LVEDD decrease after 6 months of metoprolol treatment (from 65 ± 13 mm to 63 ± 12 mm) compared to those with the A/A genotype (Ser/Ser), who showed an LVEDD increase (from 61 ± 900 mm to 63 ± 90 mm).	No	*p =* 0.030	A (Ser): lower G (Gly): higher
Magvanjav O et al. (2017) [36]	Retrospective study	926 hypertensive patients	Not specified	MCE	Patients with the G/G (Gly/Gly) or G/A (Ser/Gly) genotype showed a higher cumulative incidence of MCE compared to those with A/A (Ser/Ser) genotype (15.70% vs. 7.60%).	No	*p* = 0.018	A (Ser): higher G (Gly): lower

SBP: systolic blood pressure; HR: heart rate; LVEDD: left ventricular end-diastolic diameter; ACEI: angiotensin-converting enzyme inhibitor; ARBs: angiotensin receptor blockers; MCE: major cardiovascular events; RCT: randomized controlled trial; receptor blockade: the degree of β-receptor blockade.

**Table 4:** Efficacy outcomes, degree of receptor blockade, and evidence levels for *ADRB1* rs1801252 (g.5231 A>G; Ser49Gly) variant.

Outcome	Total Number of Articles	Outcome Variation Associated with Higher Receptor Blockade	Enhanced Receptor Blockade with A Allele (Ser)	Enhanced Receptor Blockade with G Allele (Gly)	Level of Evidence
SBP	2	SBP reduction	[19,22]		Moderate (2:0)
HR	1	HR reduction	[24]		Low (1:0)
LVEDD	1	LVEDD reduction		[35]	Low (0:1)
MCE	1	Lower incidence	[36]		Low (1:0)
Overall	5		4	1	High (4:1)

SBP: systolic blood pressure; HR: heart rate; LVEDD: left ventricular end-diastolic diameter; MCE: major cardiovascular events.

**Table 5:** Association between *ADRB2* rs1042714 (g.5318 G>C; p.Glu27Gln) and β-blocker efficacy and safety.

Author (Year)	Study Type	Population	Drug	Outcome	Association	Genotyping of *CYP2D6*	Significance	Higher/Lower Blockade
Efficacy								
Laccarino G et al. (2005) [37]	Prospective study	1050 hypertensive patients	Atenolol and metoprolol (combination with Statin, fibrates, diuretics, ACEi, and ARBs)	Dyslipidemia	Patients with the G/G genotype (Glu/Glu) showed a higher incidence of dyslipidemia compared to those with the C/C genotype (Gln/Gln) (48.40% vs. 37.30%).	No	*p* < 0.050	NA
				TG levels	Patients with the G/G genotype (Glu/Glu) showed higher serum TG levels compared to those with patients with the G/C Glu/Gln or C/C (Gln/Gln) genotypes (Gln/Gln: 12.90%; Gln/Glu: 18.60%; Glu/Glu: 25.00%).		*p* < 0.020	NA
Isaza C et al. (2005) [43]	Prospective study	141 healthy volunteers	Propranolol (monotherapy)	HDL-C levels	Volunteers with the C/C genotype (Gln/Gln) showed lower propranolol-induced HDL-C levels (baseline: 37.80 ± 4.40 mg/dL vs. post-propranolol: 31.40 ± 6.20 mg/dL) compared to those with the G/G genotype (Glu/Glu) (baseline: 42.30 ± 18.90 mg/dL vs. post-propranolol: 40.00 ± 19.30 mg/dL).	No	*p* = 0.005	NA
				TG levels	Volunteers with the G/G genotype (Glu/Glu) showed higher propranolol-induced TG levels (baseline: 119.80 ± 85.90 mg/dL vs. post-propranolol: 242.30 ± 179.80 mg/dL) compared to those with the C/C genotype (Gln/Gln) (baseline: 173.00 ± 105.60 mg/dL vs. post-propranolol: 169.10 ± 97.30 mg/dL).		*p* = 0.012	NA
Isaza CA et al. (2007) [38]	Prospective study	105 hypertensive patients	Metoprolol (monotherapy)	TC levels	Patients with the C/C genotype (Gln/Gln) showed lower TC levels during metoprolol treatment (pretreatment: 217 ± 45 mg/dL, during treatment: 208 ± 41 mg/dL), with no changes in patients with the G/C genotype (Glu/Gln) (pretreatment: 199 ± 32 mg/dL, during treatment: 206 ± 42 mg/dL).	No	*p* = 0.030	NA
				TG levels	Patients with the G/C genotype (Glu/Gln) showed lower TG levels with metoprolol therapy (pretreatment: 199 ± 55 mg/dL during treatment: 260.00 ± 7.10 mg/dL), with no changes in patients with the C/C genotype (Gln/Gln) (pretreatment: 215 ± 132 mg/dL, during treatment: 212 ± 148 mg/dL).		*p* = 0.025	NA
Shahin MH et al. (2019) [44]	RCT	768 hypertensive patients	Atenolol and metoprolol (combination with hydrochlorothiazide and amlodipine)	HR	Patients with the G/G genotype (Glu/Glu) showed a greater HR reduction in response to atenolol and metoprolol compared to those with the G/C (Glu/Gln) or C/C (Gln/Gln) genotype (numeric values not provided).	Yes	β = −0.83 *p* = 0.010 (atenolol); β = −1.59 *p* = 0.0007 (metoprolol)	C (Gln): lower G (Glu): higher
		368 hypertensive patients						
Kaye DM et al. (2003) [39]	Prospective study	80 patients with HF	Carvedilol (combination with ACEi, diuretics, and digoxin)	LVEF	Patients with the G/G (Glu/Glu) or G/C (Glu/Gln) genotype showed higher improvement in LVEF compared to those with the C/C (Gln/Gln) genotype (36% vs. 26%).	No	*p* = 0.003	C (Gln): lower G (Glu): higher
Metra et al. (2010) [27]	Prospective study	183 patients with HF	Carvedilol (ACEi, diuretics, or aldosterone antagonists)	LVEF	Patients with the G/G genotype (Glu/Glu) showed a larger LVEF increase compared to patients with the C/C (Gln/Gln) or G/C (Glu/Gln) genotype, considered together or separately (G/G: +13.00 ± 12.20%, C/C: +7.10 ± 8.10%, G/C: +8.30 ± 11.40%, G/C + C/C: +7.60 ± 9.60% units).	No	*p* = 0.022	C (Gln): lower G (Glu): higher
				Decline in pulmonary wedge pressure	Patients with the G/G genotype (Glu/Glu) showed a larger decline in pulmonary wedge pressure compared to patients with the G/C (Glu/Gln) or C/C (Gln/Gln) genotype at rest (G/G: −10 ± 10 mm Hg, G/C: −6 ± 10 mm Hg, C/C: −5 ± 7 mm Hg) and at peak exercise (G/G: −12 ± 9 mm Hg, G/C:−7 ± 10, C/C: −5 ± 7 mm Hg).		*p* = 0.027 (rest); *p* = 0.015 (exercise)	G (Gln): lower G (Glu): higher
Safety								
Guerra LA et al. (2022) [34]	Retrospective study	308 patients with HF	Metoprolol and Carvedilol (combination with ACEi, ARBs, or diuretics)	Survival	Patients with the G/G genotype (Glu/Glu) showed higher survival rates at higher β-blocker doses compared to those with the C/C genotype (Gln/Gln) (0.85 vs. 0.65).	No	*p* = 0.030	NA
Lanfear DE et al. (2005) [45]	Prospective study	735 patients with acute coronary syndrome	Metoprolol (combination with aspirin, ACEi or ARBs, statins, nitrates, and diuretics)	Survival	Patients with the G/C (Glu/Gln) or G/G (Glu/Glu) genotypes showed higher survival rates compared to those with C/C genotype (Gln/Gln) (3-year Kaplan–Meier death rates: 6%, 11%, and 16%, respectively).	No	*p* = 0.030	NA

HDL: high-density lipoprotein; TG: triglyceride; TC: total cholesterol; LVEF: left ventricular ejection fraction; HR: heart rate; HF: heart failure; ACEI: angiotensin-converting enzyme inhibitor; ARBs: angiotensin receptor blockers; RCT: randomized controlled trial; receptor blockade: the degree of β-receptor blockade. Dyslipidemia was excluded from this analysis since these metabolic effects are not mediated by ADRB blockade. Survival was excluded from this analysis since it was considered a clinical outcome too broad to analyze its relationship with receptor blockade.

**Table 6:** Efficacy outcomes, degree of receptor blockade, and evidence levels for *ADRB2* rs1042714 (g.5318 G>C; p.Glu27Gln) variant.

Outcome	Total Number of Articles	Outcome Variation Associated with Higher Receptor Blockade	Enhanced Receptor Blockade with G Allele (Glu) as Higher Blockade	Enhanced Receptor Blockade with C Allele (Gln)	Level of Evidence
HR	1	HR reduction	[44]		Low (1:0)
LVEF	2	LVEF increase	[27,39]		Moderate (2:0)
Pulmonary wedge pressure	2 *	Pulmonary wedge pressure reduction	[27]		Moderate (2:0)
Overall	5		5	0	Moderate ^$^ (5:0)

HR: heart rate; LVEF: left ventricular ejection fraction. Dyslipidemia was excluded from this analysis since these metabolic effects are not mediated by ADRB blockade. Survival was excluded from this analysis since it was considered a clinical outcome too broad to analyze its relationship with receptor blockade. * two independent results are presented in the same article; thus, the corresponding article was counted twice (Metra et al. (2010) [27]). ^$^ due to the limited literature available for this variant, the overall level of evidence was downgraded to moderate.

**Table 7:** Association between *ADRB2* rs1042713 (g.5285 G>A; p.Gly16Arg) and β-blocker efficacy and safety.

Author (Year)	Type of Study	Population	Drug	Outcome	Association	Genotyping of *CYP2D6*	Significance	Receptor Blockade
Efficacy								
Shahin MH et al. (2019) [44]	RCT	757 hypertensive patients	Atenolol and metoprolol (combination with hydrochlorothiazide and amlodipine)	HR	Patients with the A/A genotype (Arg/Arg) showed higher HR lowering response to atenolol compared to those with the G/A (Gly/Arg) or G/G (Gly/Gly) genotype (numeric values not provided).	Yes	β = −0.70 *p* = 0.040	G (Gly): lower A (Arg): higher
		368 hypertensive patients			Patients with the A/A genotype (Arg/Arg) showed higher HR lowering response to atenolol compared to those with the G/A (Gly/Arg) or G/G (Gly/Gly) genotype (numeric values not provided).		β = −1.15 *p* = 0.030	G (Gly): lower A (Arg): higher
Safety								
Lanfear DE et al. (2005) [45]	Prospective study	735 patients with acute coronary syndrome	Metoprolol (combination with aspirin, ACEi, ARBs, statins, nitrates, and diuretics)	ACM	Patients with the A/A genotype (Arg/Arg) showed higher death rates compared to those with the G/A (Gly/Arg) and G/G (Gly/Gly) genotypes (20% vs. 10%).	No	*p* = 0.005	NA

HR: heart rate; ACM: all-cause mortality; ACEI: angiotensin-converting enzyme inhibitor; ARBs: angiotensin receptor blockers; RCT: randomized controlled trial; receptor blockade: the degree of β-receptor blockade. All-cause mortality was excluded from this analysis since it was considered a clinical outcome too broad to analyze its relationship with receptor blockade.

**Table 8:** Efficacy outcomes, degree of receptor blockade, and evidence levels for *ADRB2* rs1042713 (g.5285 G>A; p.Gly16Arg) variant.

Outcome	Total Number of Articles	Outcome Variation Associated with Higher Receptor Blockade	Enhanced Receptor Blockade with A Allele (Arg)	Enhanced Receptor Blockade with G Allele (Gly)	Level of Evidence
HR	2 *	HR reduction	[44]		Moderate (2:0)
Overall	2		2	0	Low ^$^ (2:0)

HR: heart rate. * two independent results are presented in the same article; thus, the corresponding article was counted twice (Shahin MH et al. (2019) [44]). All-cause mortality was excluded from this analysis since it was considered a clinical outcome too broad to analyze its relationship with receptor blockade. ^$^ due to the limited literature available for this variant, the overall level of evidence was downgraded to low.

## Data Availability

Not applicable.
